# P-1989. Utility of Karius Testing in High-Risk Immunocompromised Hosts: A Retrospective Study

**DOI:** 10.1093/ofid/ofaf695.2156

**Published:** 2026-01-11

**Authors:** Divisha Sharma, Drew W Charles, Courtney E Harris, Susan E Dorman, Alexandra G Mills, Yosra Alkabab, Charles Teixeira, Eric G Meissner, Scott R Curry, Ruth O Adekunle, Logan Patterson, Rachel Burgoon

**Affiliations:** Ochsner Health, New Orleans , LA; Medical University of South Carolina, Charleston, South Carolina; Medical University of South Carolina, Charleston, South Carolina; Medical University of South Carolina, Charleston, South Carolina; Medical University of South Carolina, Charleston, South Carolina; MUSC, Charleston, South Carolina; MUSC, Charleston, South Carolina; Medical University of South Carolina, Charleston, South Carolina; Medical University of South Carolina, Charleston, South Carolina; Medical University of South Carolina, Charleston, South Carolina; MUSC, Charleston, South Carolina; Medical University of South Carolina, Charleston, South Carolina

## Abstract

**Background:**

Plasma metagenomic next-generation sequencing or the Karius test (KT) can simultaneously detect hundreds of pathogens, yet real-life performance data in heterogeneous immunocompromised (IC) adults remain limited. We examined KT utilization, diagnostic yield, turnaround time, and downstream clinical consequences in IC patients—including human immunodeficiency virus (HIV), solid-organ transplant (SOT), hematopoietic stem-cell transplant (HSCT), and those with active hematologic or solid malignancies—managed at a tertiary center.

**Figure:**
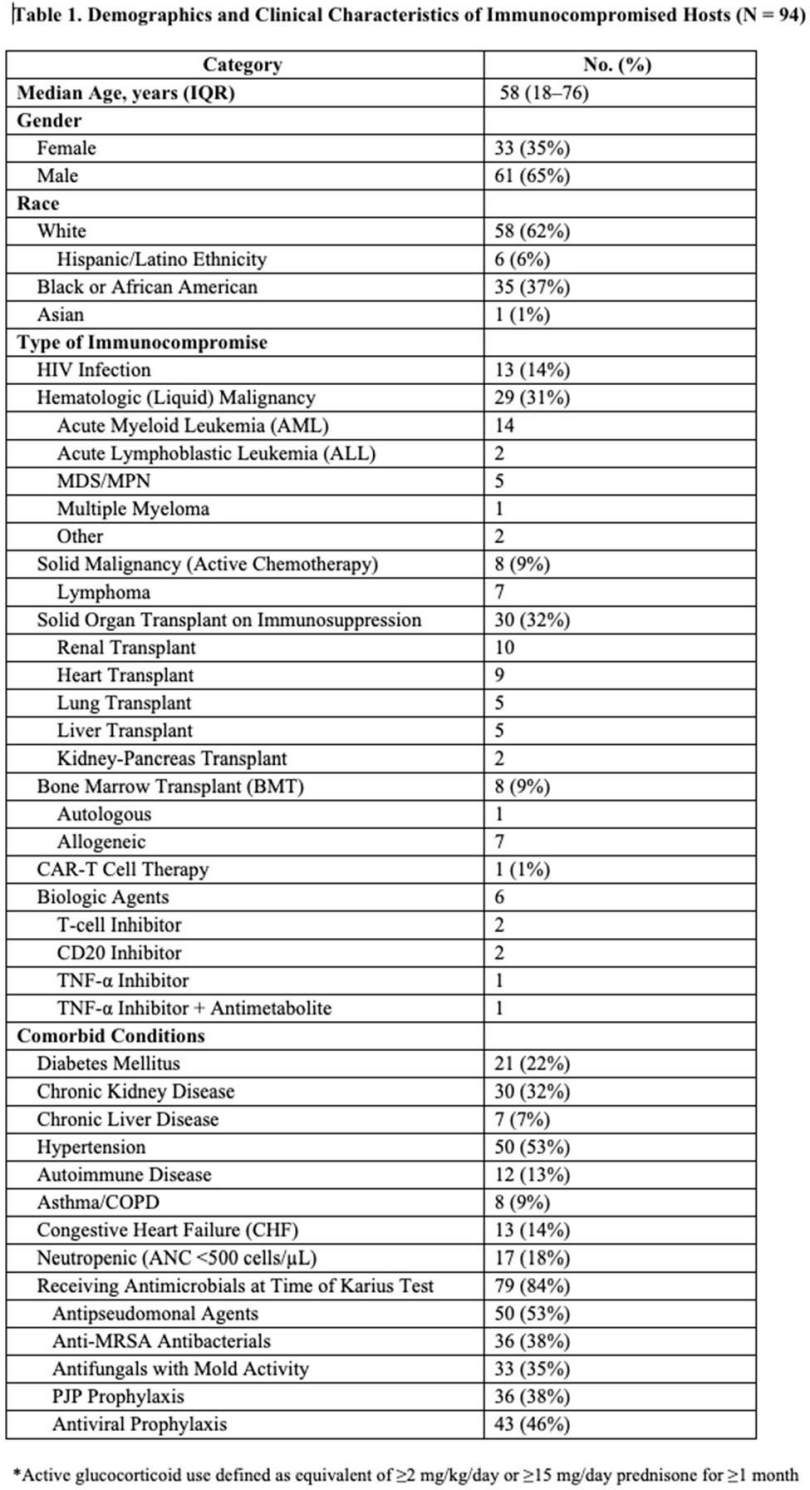

**Figure:**
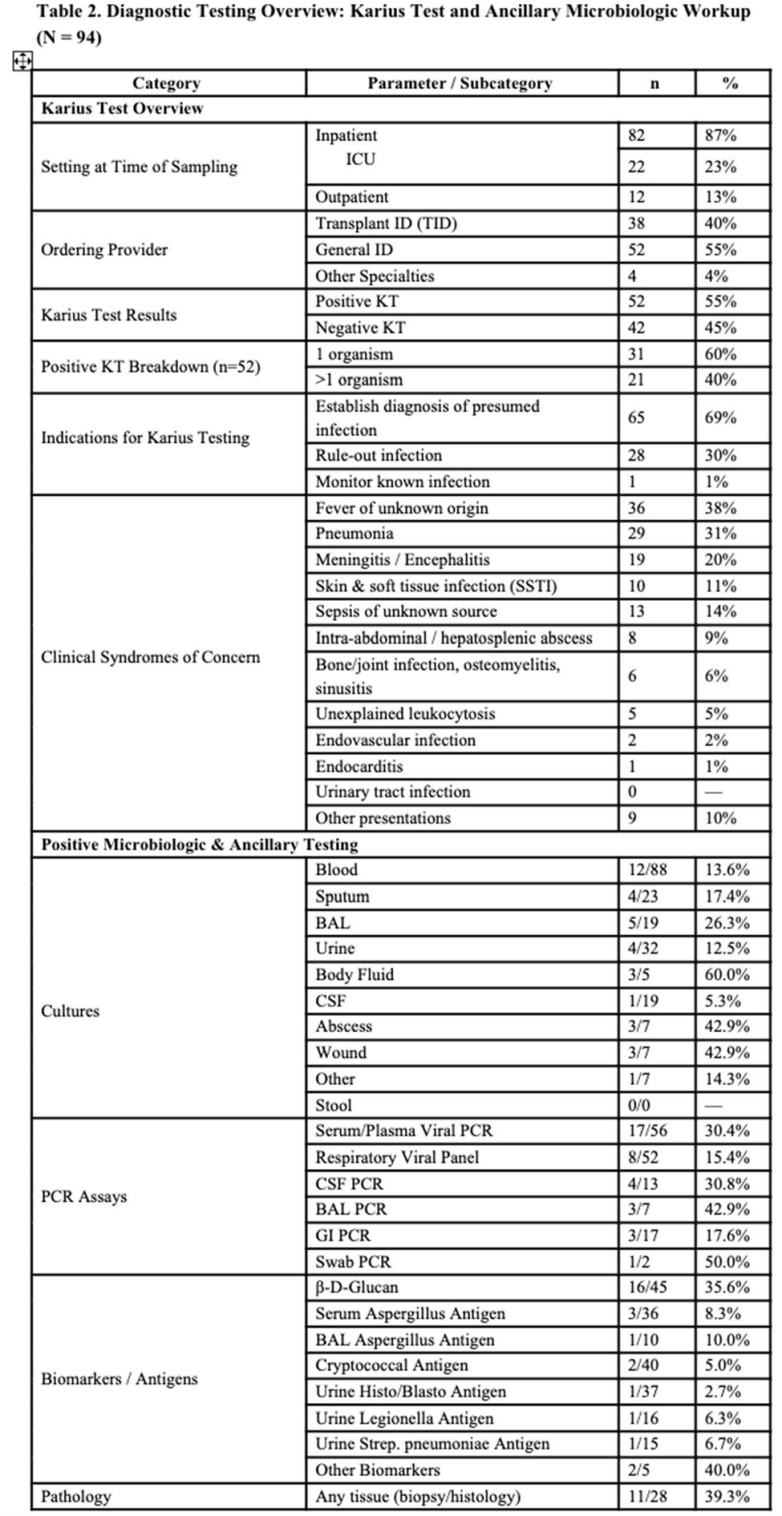

**Methods:**

This single-center retrospective study included all adults who underwent KT from January 1, 2022, through November 30, 2024. Demographics, immunosuppressive condition, antimicrobial exposure, indication for testing, and conventional microbiology were extracted from medical records.

**Figure:**
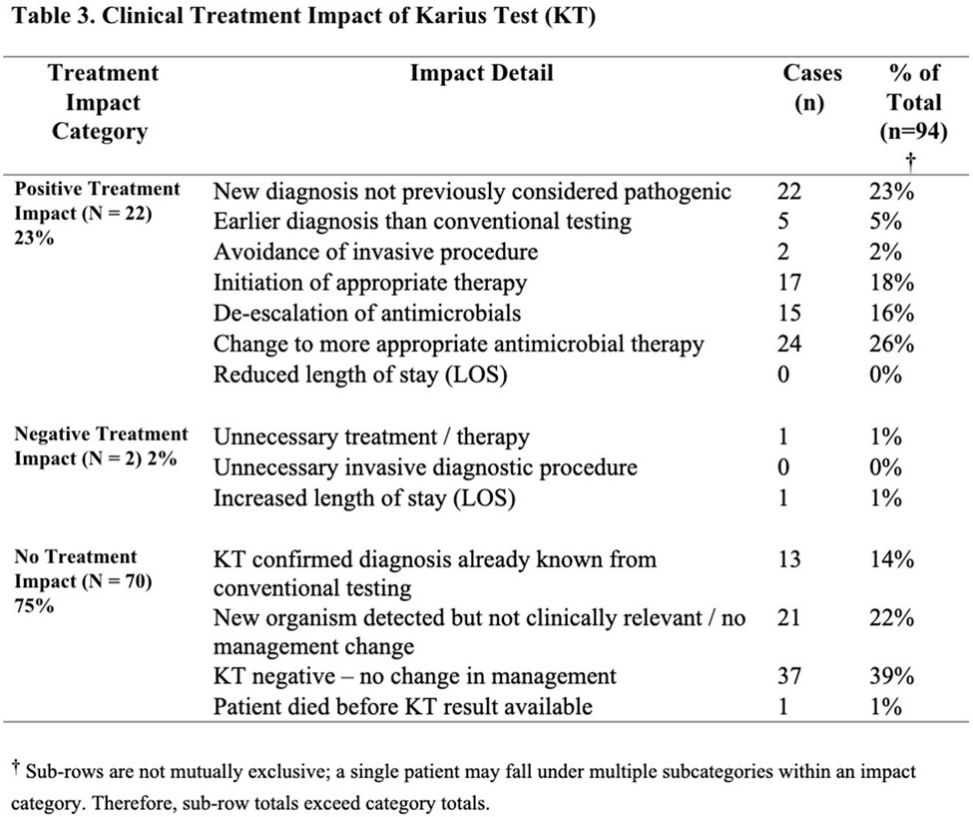

**Figure:**
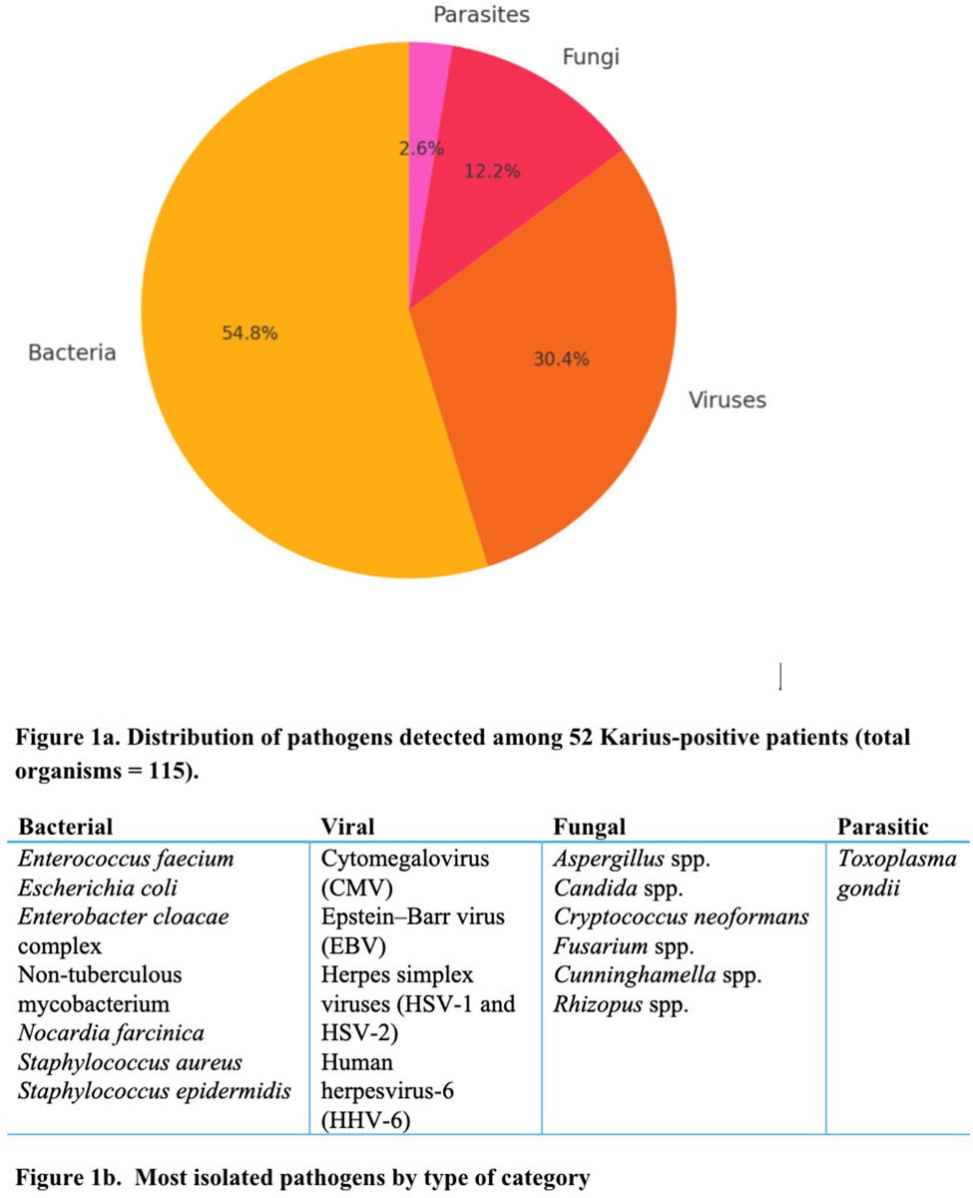

**Results:**

Ninety-four IC patients were identified with demographics outlined in Table 1. Of those, 62% had a SOT, 22% had a HSCT, and 16% had diagnosed malignancy without transplant. At sampling, over 80% were receiving antimicrobials and were hospitalized. The indication for KT was to establish a diagnosis for a problem that was presumed to be infectious in 69%, rule-out infection in 30%, or monitor known infection in 1% (Table 2). Most common organisms isolated are displayed in Figure 1. KT was positive in 52 cases (55%): with a mean KT turnaround of 3.3 days (IQR 2-8) .Positive clinical treatment impact occurred in 22 cases (23%) by identifying a previously unsuspected pathogen, accelerating diagnosis, or optimizing antibacterial or antifungal therapy; 2 cases (2%) had negative impact, and 70 cases (75%) were neutral (Table 2). KT provided actionable information in 40% of culture-negative/PCR-negative work-ups. All-cause 90-day mortality was 22%.

**Conclusion:**

In a diverse IC cohort, KT produced clinically meaningful changes in management for nearly one-quarter of patients while rarely causing harm. Its rapid turnaround and ability to uncover otherwise undetected pathogens support targeted use alongside conventional diagnostics. Prospective studies should define optimal patient selection and assess cost-effectiveness across IC subgroups.

**Disclosures:**

Rachel Burgoon, Pharm.D., Merck: Grant/Research Support

